# A novel vascular model yields increased MR perfusion metrics compared to conventional dynamic susceptibility contrast algorithms in untreated glioblastoma

**DOI:** 10.1093/noajnl/vdaf212

**Published:** 2025-09-30

**Authors:** Jonas Reis, Robert Stahl, Katharina J Müller, Philipp Karschnia, Nico Teske, Antonia Neubauer, Louisa von Baumgarten, Niklas Thon, Florian Ringel, Thomas Liebig, Nathalie L Albert, Patrick N Harter, Robert Forbrig

**Affiliations:** Institute of Neuroradiology, LMU University Hospital, LMU Munich, Munich, Germany; Institute of Neuroradiology, LMU University Hospital, LMU Munich, Munich, Germany; Department of Neurology, LMU University Hospital, LMU Munich, Munich, Germany; Department of Neurosurgery, Universitaetsklinikum Erlangen, Friedrich-Alexander-University, Erlangen-Nuremberg, Germany; Department of Neurosurgery, Universitaetsklinikum Erlangen, Friedrich-Alexander-University, Erlangen-Nuremberg, Germany; Center for Neuropathology and Prion Research, Faculty of Medicine, LMU Munich, Munich, Germany; Department of Neurology, LMU University Hospital, LMU Munich, Munich, Germany; Department of Neurosurgery, LMU University Hospital, LMU Munich, Munich, Germany; German Cancer Consortium (DKTK), Partner Site Munich, Munich, Germany; Bavarian Cancer Research Center (BZKF), Munich, Germany; Department of Neurosurgery, Knappschaft University Hospital, RUB, Bochum, Germany; Department of Neurosurgery, LMU University Hospital, LMU Munich, Munich, Germany; Institute of Neuroradiology, LMU University Hospital, LMU Munich, Munich, Germany; Department of Nuclear Medicine, LMU University Hospital, LMU Munich, Munich, Germany; German Cancer Consortium (DKTK), Partner Site Munich, Munich, Germany; Bavarian Cancer Research Center (BZKF), Munich, Germany; Center for Neuropathology and Prion Research, Faculty of Medicine, LMU Munich, Munich, Germany; German Cancer Consortium (DKTK), Partner Site Munich, Munich, Germany; Bavarian Cancer Research Center (BZKF), Munich, Germany; Institute of Neuroradiology, LMU University Hospital, LMU Munich, Munich, Germany

**Keywords:** Bayesian modeling, glioblastoma, gradient echo dynamic susceptibility contrast perfusion, magnet resonance imaging, neoangiogenesis

## Abstract

**Background:**

Malignant gliomas are heterogeneous brain tumors with extensive neovascularization. Conventional gradient-echo dynamic susceptibility contrast (GRE-DSC) perfusion MRI may underestimate microvascular alterations. We hypothesized that a novel vascular model (NVM), based on Bayesian voxel-wise transit time distribution analysis, could yield higher perfusion metrics in untreated isocitrate dehydrogenase (*IDH*)-wild-type glioblastoma compared to standard vendor GRE-DSC algorithms.

**Methods:**

In this retrospective, single-center study, 89 patients with neuropathologically confirmed glioblastoma underwent pretherapeutic GRE-DSC perfusion MRI at 1.5 or 3.0 T. Perfusion maps were generated using both the NVM and default vendor algorithms. Using co-registered T1-post-contrast and T2/FLAIR images, two neuroradiologists independently assessed perfusion conspicuity of color-coded maps for each algorithm and manually performed region-of-interest analyses within visually identified tumor hotspots for quantification. Relative values of cerebral blood flow (rCBF), cerebral blood volume (rCBV), and mean transit time (rMTT) were normalized to contralateral normal-appearing white matter. Nonparametric tests evaluated group differences.

**Results:**

The NVM yielded enhanced hotspot delineation and significantly higher median normalized perfusion values than vendor algorithms (all *P* < .001), with excellent inter-rater reliability (Cohen’s κ and intraclass correlation coefficients ≥0.86). At 3.0 T, NVM-derived rCBV was significantly higher than at 1.5 T (*P* = .008).

**Conclusions:**

NVM post-processing yielded higher normalized CBF, CBV, and MTT values within tumor hotspots than vendor pipelines, suggesting that Bayesian model-based perfusion analysis may enhance the detection of microvascular changes in glioblastoma. As validation against a gold standard is missing, prospective multicenter studies are warranted to confirm our findings, particularly with regard to treatment monitoring and clinical decision-making.

Key Points

- NVM may enhance visual delineation of MR perfusion conspicuity in untreated glioblastoma.
- NVM yielded significantly higher metrics in tumor hotspots indicating increased detection of microvascular changes.
- Differences in perfusion metrics were highest at 3.0 T.

Importance of the Study

Gradient-echo dynamic susceptibility contrast perfusion MRI is usually post-processed with singular-value decomposition (SVD), a method that can underestimate the microvascular heterogeneity inherent to malignant gliomas. In this study of untreated *IDH*-wild-type glioblastoma, we compared a Bayesian Novel Vascular Model (NVM), which fits a voxel-wise capillary transit-time distribution, with vendor-based standard SVD processing. NVM yielded consistently higher CBF, CBV, and MTT metrics of tumor hotspots, suggesting improved depiction of tumor hyperperfusion. Prospective, multicenter studies with an independent gold standard are now needed to confirm that these numerical gains truly reflect microvascular biology.

Magnetic resonance imaging (MRI), including dynamic susceptibility contrast (DSC) perfusion, is a cornerstone in the diagnosis and monitoring of glioblastoma as it provides detailed insights such as tumor morphology, vascularization, metabolism, and treatment response. DSC perfusion applies the so-called indicator-dilution theory, interpreting the first-pass T_2_ signal drop after an intravascular gadolinium bolus as a surrogate concentration-time curve.^1^^,^^2^ Among available MRI perfusion techniques, gradient-echo DSC (GRE-DSC) is most widely used to quantify cerebral blood flow (CBF), cerebral blood volume (CBV) and mean transit time (MTT).^3^ Relative CBV (rCBV) is particularly valuable: elevated values correlate with micro-vascular density, tumor grade and molecular characteristics as well as patient prognosis^4-7^ and help to distinguish true progression from treatment-related changes.^8^^,^^9^

Conventional GRE-DSC post-processing relies on singular-value-decomposition (SVD) deconvolution, which is delay-sensitive^10^ and prone to oscillatory residue functions of limited physiological plausibility.^11^ To overcome both acquisition and analysis shortcomings, a Bayesian Novel Vascular Model (NVM) was developed that fits a gamma-distributed capillary transit-time residue and outputs capillary-transit-time heterogeneity (CTH) from core GRE-DSC data.^12^ CTH is clinically relevant because widening of the transit-time distribution impairs oxygen extraction and can precipitate ‘malignant’ micro-vascular shunting.^13^

Thus—alongside CBF, CBV, and MTT—the NVM is particularly capable to generate oxygenation-related metrics (eg oxygen-extraction fraction [OEF] and cerebral metabolic rate of oxygen [CMRO_2_]) from CTH. These metrics have shown promise in stroke and glioma imaging,^12^^,^^13^ and may help discriminate true glioblastoma progression from pseudoprogression or differentiate de novo tumors from recurrent lesions.^14^^,^^15^

The primary objective of this study is to directly compare NVM-derived CBF, CBV and MTT measurements of tumor hotspots with standard vendor algorithm estimates in untreated *IDH*-wild-type glioblastoma patients. We hypothesize that these perfusion metrics differ systematically of each other, reflecting the NVM’s proposed enhanced ability to detect microvascular perfusion abnormalities.

## Material and Methods

### Patients

**Figure 1.**
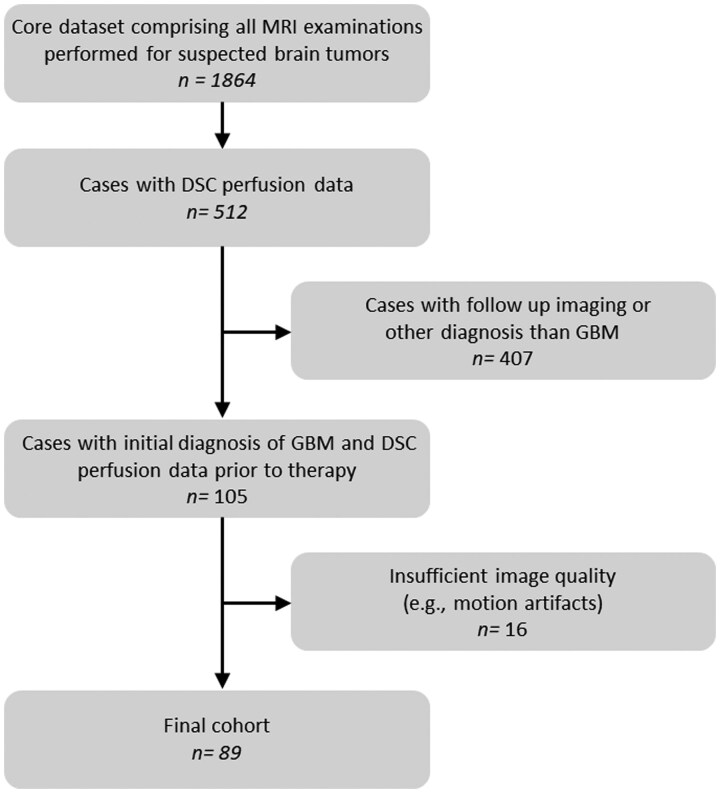
Flowchart of the patient selection process for this retrospective study. The diagram shows how 89 patients with untreated *IDH*-wildtype glioblastoma were identified, screened for eligibility, and ultimately included or excluded based on predefined criteria (eg motion artifacts, missing hyperperfusion). DSC = dynamic susceptibility contrast; GBM = glioblastoma; n = number.

In this retrospective, single-center study we analyzed GRE-DSC perfusion MRI datasets of patients with first diagnosis of *IDH*-wild-type glioblastoma acquired between January 2019 and September 2024. Regarding patient selection, a database was created by merging the results of a PACS full-text and sequence search, respectively. The resulting patient pool was then filtered by cross-referencing clinical and histological data. Only patients with histologically confirmed and/or molecularly characterized *IDH*-wild-type glioblastoma who underwent GRE-DSC perfusion MRI prior to any therapeutic intervention were included. Neuropathologically, molecular characterization was performed according to the latest WHO CNS tumor classification.^16^ Out of the resulting cases, datasets exhibiting motion artifacts, absence of hyperperfusion in the target regions, or other substantial imaging distortions were excluded. Detailed inclusion and exclusion criteria are presented in Figure 1.

### Imaging Acquisition

MRI was performed on three scanners: Signa HD (3 Tesla (T); GE Healthcare, Illinois, USA), Magnetom Vida (3 T; Siemens Healthineers, Erlangen, Germany) and Magnetom Sola Fit (1.5 T; Siemens Healthineers). The standard protocol comprised T2 and T2-FLAIR (fluid-attenuation inversion recovery) sequences as well as non-contrast and contrast-enhanced T1-weighted imaging. GRE-DSC perfusion was acquired with the vendor-recommended tumor protocols summarized in Table 1. All GRE-DSC runs used gadobutrol (1.0 mmol ml^−1^; Gadovist, Bayer Vital, Leverkusen, Germany) with a 0.05 mmol kg^−1^ preload bolus followed by a 0.1 mmol kg^−1^ dose at 3-5 ml s^−1^, chased by 20 ml saline.

**Table 1.** GRE-DSC perfusion acquisition parameters

Scanner	TR (ms)	TE (ms)	Flip angle (°)	Temporal resolution (s)
GE Signa HD	1400	20	60	1.40
Siemens Magnetom Vida	1600	30	90	1.60
Siemens Magnetom Sola fit	2770	31	90	2.77

All scans employed a gadobutrol (Gadovist) bolus (0.1 mmol kg^−1^ dose at 3-5 ml s^−1^) preceded by a 0.05 mmol kg^−1^ preload to reduce T_1_ leakage effects, followed by 20 ml saline. Flip angle, temporal resolution, and TR/TE settings reflect each vendor’s recommended tumor-perfusion protocol for the specified field strength.

Abbreviations: GRE-DSC, gradient-echo dynamic susceptibility contrast; TE, echo time; TR, repetition time.

### Vendor Perfusion Pipelines

GE BrainStat (SIGNA Works DV26-29, AW 4.6) automatically averages early arterial voxels into one global arterial input function (AIF), then deconvolves all tissue curves with a block-circulant SVD (oSVD) kernel.^10^ A post-fit baseline restoration yields a leakage-indicator map (K_2_) and nominal rCBV/rCBF/rMTT; only CBV is partly corrected for T_1_/T_2_* tailing.^17^ Siemens syngo. MR Neuro Perfusion (XA50/60) derives a slice-specific local AIF and applies the same oSVD kernel. It produces rCBV/rCBF/rMTT maps without leakage correction.

### NVM Perfusion Pipeline

The NVM pipeline (Neurosuite, v16.1, Cercare Medical, Aarhus, Denmark) first selects a single global arterial-input function automatically and applies it voxel-wise.^11^ Rather than SVD deconvolution, each voxel’s concentration-time curve is fitted with a physiologically constrained gamma residue using a Bayesian expectation-maximization algorithm.^11^^,^^12^ The fit updates CBF, MTT, macrovascular delay and noise variance; CBV is then computed as CBF × MTT. A co-estimated, unidirectional leakage term (*K*_app_) removes T_1_/T_2_* tailing before the fit, yielding leakage-corrected rCBV, rCBF and rMTT maps; ^18^ To note, *K*_app_ is an empirical correction, not a pharmacokinetic *K*_trans_.

### Qualitative Analysis

By matching the T2/FLAIR as well as non-contrast and contrast-enhanced T1-weighted sequences, qualitative hyperperfusion hotspot conspicuity was assessed independently by two board-certified neuroradiologists (6- and 12-years’ experience in diagnostic neuroradiology). Color-encoded maps (rCBV, rCBF, rMTT, respectively) were exported with the native window/level presets of each pipeline, displayed on identical calibrated monitors, renamed with random numeric identifiers, and presented side-by-side in random order to reduce reader recognition of the processing method. A three-point ordinal scale was applied: 0 = inadequate hyperperfusion hotspot delineation, 1 = adequate hyperperfusion hotspot delineation, 2 = good hyperperfusion hotspot delineation.

### Quantitative Analysis

**Figure 2.**
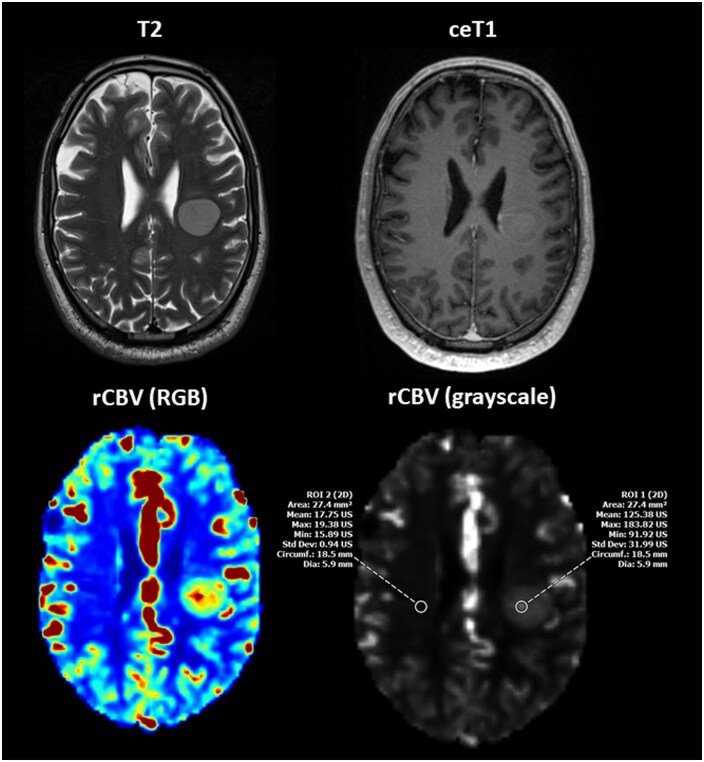
Example of ROI placement in a 42-year-old male with newly diagnosed left subcentral glioblastoma (MGMT-methylated, TERT C228T mutation), scanned at 3.0 T. Axial T2-weighted (T2) and contrast-enhanced T1-weighted (ceT1) images show a well-delineated, homogeneous lesion with minor rim enhancement. The NVM-derived rCBV (RGB) and rCBV (grayscale) maps illustrate ROI placement: ROI 1 is located within the perfusion hotspot identified on the color-coded (RGB) rCBV map, while ROI 2 is placed in contralateral normal-appearing white matter, each ROI measuring 22.4 mm^2^. Despite initially atypical MRI morphology, histology revealed the characteristic features of a malignant glioma. Six-week follow-up imaging (not shown) demonstrated typical rim enhancement and central necrosis as well as mild perifocal edema. ROI = region of interest; rCBV = relative cerebral blood volume; RGB = red-green-blue.

For quantitative hyperperfusion hotspot analysis, two board-certified neuroradiologists (6- and 12-years’ experience in diagnostic neuroradiology) independently performed manual region of interest (ROI) placement (circular, 20–30 mm^2^) on every grey-scale perfusion map (rCBF, rCBV, rMTT, respectively) under evaluation.^19^ Color-coded maps and co-registered T1-post-contrast and T2/FLAIR images were used to refine the placement (Figure 2). Macroscopically visible vessels and necrotic areas were excluded to avoid perfusion inflation. An identically sized control ROI was placed in contralateral normal-appearing white matter (NAWM). Datasets were displayed in randomized order with anonymized file names, reducing pipeline recognition. Normalized metrics were calculated per map as nMetric = rMetric(hotspot)/rMetric(NAWM) for CBF, CBV, MTT, respectively. Mean normalized values were then entered into predefined comparisons:

1. NVM vs vendor (all scanners and field strengths).
2. NVM vs vendor at different field strengths (at 3.0 T and 1.5 T, respectively).
3. Siemens scanner vs GE scanner (at 3.0 T and NVM processing only).
4. Siemens pipeline vs GE pipeline (at 3.0 T)

### Statistics

Inter-rater reliability was evaluated for qualitative analyses by application of Cohen’s kappa and quantitative analyses by application of consistency- and agreement-based intraclass correlation coefficients (ICC). The normality of continuous data was assessed using the Shapiro–Wilk test. As most variables did not follow a normal distribution, descriptive statistics are reported as medians and interquartile ranges (25th and 75th percentiles). Non-parametric tests were used for group comparisons: the Wilcoxon signed-rank test for paired comparisons between NVM and vendor algorithms, and the Mann-Whitney U test for independent samples comparing scanner field strengths and manufacturers. For binary variables (eg sex) and categorical variables (eg distribution of molecular biomarkers), contingency tables were created. The independence of these variables was assessed using the Chi-squared test or Fisher’s exact test, as appropriate. Statistical analyses were performed using R (version 4.3.2, R Core Team, 2023) with a significance level of α  =  .05.

## Results

In total, 89 patients (37 female, mean age 66.0 years) with first diagnosis of *IDH*-wild-type glioblastoma were analyzed, with most scans acquired on 3.0 T systems (74/89 patients, 83%) and an approximately equal distribution between GE and Siemens scanners (45 vs. 44 patients). Of the assessed molecular markers, a MGMT promoter methylation was documented in 31/89 (34.8%) patients and a TERT promoter mutation in 76/89 (85.4%) patients. Further patient and neuropathological characteristics are provided in Table 2.

**Table 2.** Patient and neuropathological characteristics

Age (years)	66.0 ± 11.9 (39-89)^a^
Sex	
Female	37 (41.6%)^b^
Male	52 (58.4%)^b^
TERT promoter	
C228T mutation	51 (57.3%)^b^
C250T mutation	25 (28.1%)^b^
no mutation	11 (12.4%)^b^
n.a.	2 (2.2%)^b^
MGMT promoter	
No/partial methylation	58 (65.2%)^b^
methylation	31 (34.8%)^b^
Ki-67 proliferation index^c^	
0	3 (3.4%)^b^
1	21 (23.6%)^b^
2	55 (61.8%)^b^
3	10 (11.2%)^b^
Neuropathological diagnosis	
Histological and molecular criteria	85 (95.5%)^b^
Molecular criteria only	4 (4.5%)^b^

Abbreviations: MGMT, O6-methylguanine-DNA methyltransferase; TERT, telomerase reverse transcriptase; n.a., not available.

a Mean value ± standard deviation (range).

b Numbers (percentage).

c Ki-67 proliferation index: 0 = no or rare occurrence of staining, 1 ≤ 10% of cells positively stained, 2 = 10-30% of cells positively stained, 3 ≥ 30% of cells positively stained. Score 0-1, Score 2 and Score 3 indicated low, moderate and high expression of Ki-67.

### Qualitative and Quantitative Analysis

Detailed statistics of qualitative assessments and for each quantitative parameter are provided in Tables 3 and 4.

**Table 3.** Qualitative and quantitative results

	NVM	Vendor	*P* value^a^
Image quality			
rCBF 1^)^	2 [1; 2]	1 [0.5; 1]	**<.001**
rCBV 1^)^	2 [2; 2]	1 [1; 1]	**<.001**
rMTT 1^)^	2 [1; 2]	0 [0; 1]	**<.001**
Signal ratio			
All scanners			
nCBF (mean) 2^)^	9.96 [6.42; 19.13]	5.52 [3.16; 7.75]	**<.001**
nCBV (mean) 2^)^	8.15 [5.41; 11.33]	5.56 [3.52; 8.18]	**.001**
nMTT (mean) 2^)^	3.99 [2.57; 6.92]	1.73 [1.29; 2.19]	**<.001**
3.0 T			
nCBF (mean)3^)^	9.93 [6.44; 19.91]	5.69 [3.20; 7.97]	**<.001**
nCBV (mean)3^)^	8.54 [5.80; 11.58]	5.73 [3.54; 8.83]	**.002**
nMTT (mean)3^)^	3.96 [2.67; 7.18]	1.73 [1.29; 2.19]	**<.001**
1.5 T			
nCBF (mean)4^)^	10.83 [6.36; 16.17]	5.52 [3.48; 7.29]	**.004**
nCBV (mean)4^)^	6.31 [4.82; 7.42]	4.23 [3.50; 7.12]	.107
nMTT (mean)4^)^	4.76 [2.40; 5.91]	1.72 [1.24; 1.98]	**.001**

Data are median [25% quartile; 75% quartile].

Abbreviations: CBF, cerebral blood flow; CBV, cerebral blood volume; max, maximum; MTT, mean transit time; n, normalized; NVM, novel vascular model; r, relative; T, Tesla.

a Values in bold indicate significant intergroup difference with Wilcoxon test. Number of observations/examinations: 1^)^  *n* = 89; 2^)^  *n* = 89; 3^)^  *n* = 74; 4^)^  *n* = 15.

**Table 4.** Quantitative results of intragroup comparisons

	GE MRI^1^)	Siemens MRI^2^)	*P* value^a^
Vendor at 3.0 T			
nCBF (mean)	5.53 [2.97; 8.19]	6.17 [3.98; 7.08]	.45
nCBV (mean)	4.65 [3.15; 8.15]	6.95 [5.67; 9.57]	**.02**
nMTT (mean)	1.72 [1.29; 2.18]	1.74 [1.31; 2.52]	.59
NVM at 3.0 T			
nCBF (mean)	9.60 [4.70; 20.17]	10.39 [7.58; 19.13]	.27
nCBV (mean)	8.55 [5.77; 11.36]	8.44 [5.87; 11.58]	.92
nMTT (mean)	3.79 [2.57; 7.26]	4.50 [6.92; 17.54]	.6

Data are median [25% quartile; 75% quartile].Abbreviations: CBF, cerebral blood flow; CBV, cerebral blood volume; max, maximum; MTT, mean transit time; n, normalized; NVM, novel vascular model; r, relative; T, Tesla.

a Values in bold indicate significant intergroup difference with Wilcoxon test. Number of observations/examinations: ^1)^  *n* = 45; ^2)^  *n* = 29.

**Figure 3.**
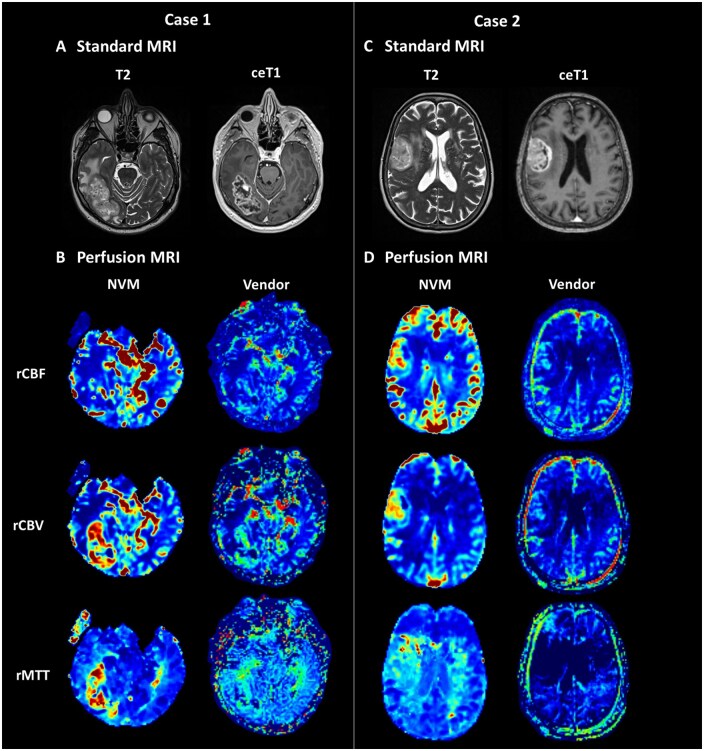
Illustrative DSC-MRI perfusion comparisons between the novel vascular model (NVM) and vendor algorithms in two glioblastoma cases. Axial T2-weighted (T2) and contrast-enhanced T1-weighted (ceT1) images in (A) a 65-year-old male with temporo-occipital glioblastoma (unmethylated MGMT, TERT C228T mutation) at 3.0 T and (C) a 74-year-old female with right frontal glioblastoma (unmethylated MGMT, TERT C228T) at 1.5 T. Tumors exhibit rim enhancement, central necrosis, and varying degrees of perifocal edema (A, C). Corresponding color-coded RGB maps for rCBF, rCBV, and rMTT derived from either the NVM or vendor algorithm (B, D). NVM maps show more pronounced hyperperfusion (rCBF, rCBV) and prolonged transit times (rMTT) with improved spatial resolution and higher SNR. In contrast, vendor maps display less distinct hyperperfusion and increased image noise, particularly for rMTT in case 2. DSC = dynamic susceptibility contrast; T = Tesla; rCBF = relative cerebral blood flow; rCBV = relative cerebral blood volume; rMTT = relative mean transit time; RGB = red-green-blue; SNR= signal-to-noise ratio.

Regarding inter-rater reliability of qualitative DSC-GRE perfusion analysis Kappa values ranged from 0.85 to 0.93 (*P* < .0001), with ratings on NVM perfusion maps being significantly higher compared to vendor perfusion maps (rCBF, rCBV, rMTT; *P* < .0001, each). Illustrative cases are shown in Figure 3.

**Figure 4.**
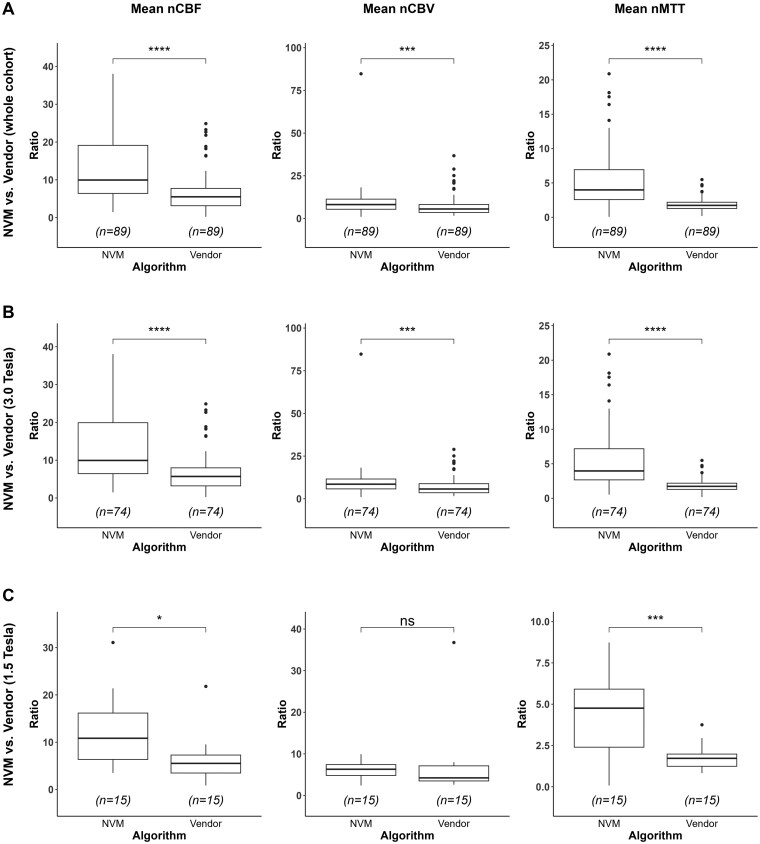
Quantitative analysis of normalized DSC-MRI perfusion parameters comparing the NVM with vendor algorithms. Box plots display nCBF, normalized cerebral blood volume nCBV, and normalized mean transit time nMTT for the whole cohort (A), 3.0 T subgroup (B), and 1.5 T subgroup (C). Asterisks indicate significance levels: * = *P* < .05, *** = *P* < .001; ns = not significant. DSC = dynamic susceptibility contrast; NVM = novel vascular model; nCBF = normalized cerebral blood flow; nCBV = normalized cerebral blood volume; nMTT = normalized mean transit time; T = Tesla.

For quantitative analysis of DSC-GRE MRI perfusion parameters, intraclass correlation coefficients ranged from 0.86 to 0.94 across consistency- and agreement-based calculations. NVM-derived ratios were significantly higher compared to those obtained from vendor algorithms (mean nCBF, mean nCBV, mean nMTT; *P *< .001, each; Figure 4A). When comparing NVM and vendor algorithms at 3.0 T, NVM-derived ratios were significantly higher (nCBF, nCBV, and nMTT, *P* < .001, each; Figure 4B). At 1.5 T, NVM-derived nCBF and nMTT ratios were significantly higher than vendor ratios (*P* < .05), whereas nCBV did not reach statistical significance (Figure 4C). Subgroup analysis of NVM at 1.5 T vs. 3.0 T showed that nCBV was significantly higher at 3.0 T (*P* = .008), whereas nCBF and nMTT remained consistent across field strengths (*P* > .05, each). At 3.0 T, intragroup comparison of the vendor pipelines showed that Siemens produced significantly higher nCBV values than GE, whereas no significant differences were detected for nCBF or nMTT. Within the NVM pipeline at 3.0 T, no significant differences in nCBF, nCBV, or nMTT were observed between the GE and Siemens scanners. Furthermore, no significant group-wise differences in demographic or molecular characteristics were identified.

## Discussion

In this retrospective cohort of 89 untreated *IDH*-wild-type glioblastomas, the NVM consistently yielded higher normalized perfusion ratios and more distinct visual hotspot delineation than the two applied vendor pipelines with excellent inter-reader agreement. The improvement was most significant for nCBF and nMTT known to be most vulnerable to delay-related under-estimation in conventional SVD processing, utilized by vendor pipelines.^10^ Importantly, the numerical gains of NVM were vendor-agnostic: similar gains were observed over both vendor pipelines, indicating that most of the benefit stems from the NVM rather than MRI platform-specific differences. These results extend previous simulations and small-series reports suggesting that conventional GRE-DSC perfusion may rather under-detect high-flow and heterogeneous transit components,^11^^,^^20^ by demonstrating a tangible clinical impact on hotspot conspicuity in a contemporary, multi-scanner glioblastoma cohort.

Previous validations of the NVM were confined to simulations and small patient samples relative to oSVD, but not benchmarked against vendor software.^11^^,^^12^ The significant elevations we observed in nCBF, nCBV, and nMTT when applying the NVM are concordant with phantom simulations and in-vivo studies demonstrating that block-circulant oSVD underestimates perfusion when the bolus is delayed or dispersed.^10^^,^^20^ We extend these theoretical and small-scale observations by demonstrating that the numerical gain translates into more distinct hot-spot conspicuity on routine clinical images, a link not previously reported.

Our intent was a pragmatic head to head comparison against conventional vendor DSC processing that reflects how GRE DSC perfusion is typically used at the scanner console and in clinical workstations. This baseline choice is directly relevant to translation and adoption. Consistent with this design, we report preload dosing, contralateral NAWM normalization, and excellent inter reader agreement, and we carefully limit claims to relative numerical differences and conspicuity rather than biological accuracy. The observation that the largest gains occurred in nCBF and nMTT, metrics comparatively less sensitive to residual leakage effects after preload, further supports that the effect is not solely explained by leakage correction.

Vendor pipelines that rely on oSVD remain vulnerable to these bolus-timing and dispersion effects, which are common in malignant gliomas.^10^^,^^21^ NVM circumvents the hard threshold by fitting a gamma-distributed residue in a Bayesian framework, recovering capillary transit components,^11^ that oSVD suppresses and thus yielding higher perfusion metrics. However, GRE-DSC perfusion scales with the square of vessel radius, probably over-representing larger vessels relative to capillaries.^22^^,^^23^ In contrast, spin-echo (SE) DSC perfusion, whose signal scales with the fourth power of radius, is inherently more capillary-specific yet suffering from lower signal-to-noise ratio (SNR) and limited brain coverage.^24^^,^^25^ Given these constraints, consensus guidelines still recommend application of GRE-DSC (instead of SE-DSC or dual-echo acquisitions) for MR perfusion imaging in glioma patients,^26^ leaving micro-vascular alterations difficult to quantify.

In this study, an explicit leakage correction was solely applied by the NVM, whereas the vendor outputs were analyzed in their nominally uncorrected form. Several aspects, however, limit the extent of resulting bias. First, we administered a standard preload, to reduce underestimation of rCBV while exerting negligible influence on CBF and MTT.^27^ Second, all perfusion metrics were normalized to contralateral NAWM to abolish leakage-related differences between corrected and uncorrected nCBV.^17^ Consistent with these precautions, the largest numerical gains occurred in nCBF and nMTT, parameters largely insensitive to leakage effects after preload. Furthermore, significantly higher nCBV observed with the uncorrected Siemens pipeline compared to the GE pipeline with baseline-restoration (at 3.0 T, respectively) undermines the assumption about the influence of leakage correction.^28^ This indicates that the principal advantage of NVM stems from its physiology-based modelling rather than from leakage correction alone, although some of the nCBV increase may reflect NVM’s additional leakage term.

The extent of NVM’s numerical gains over both vendor pipelines within tumor “hotspots” in this study was depended on the applied field strength. At 3.0 T, the NVM increased nCBV significantly relative to vendor software, whereas at 1.5 T the nCBV gain did not reach significance; nCBF and nMTT remained significantly higher at both field strengths. The most plausible explanation is the higher SNR at 3.0 T, which provides the NVM with richer information, while threshold-based oSVD benefits less.^26^ This indicates that the advantages at higher field strengths may be driven by the modelling strategy rather than by manufacturer implementation and aligns with clinical comparisons showing superior lesion conspicuity at 3.0 T relative to 1.5 T for conventional DSC sequences.^29^

Both the increased normalized perfusion ratios and improved hotspot conspicuity obtained with NVM may have several translational benefits. First, a clearer delineation of hyperperfused tumor components could refine stereotactic-biopsy targeting, potentially reducing sampling errors that limit the radiology-histology concordance (eg Ki-67).^5^ Second, advanced multiparametric MRI including GRE-DSC perfusion have shown to correlate with *IDH*-mutation and MGMT promoter methylation status in glioblastomas,^30^ reinforcing NVM’s potential beyond quantitative perfusion estimates to provide biologically relevant imaging biomarkers that may aid in noninvasive tumor classification. Third, the NVM could improve the robustness of dedicated semi-quantitative perfusion scores to further refine detection of glioma recurrence.^31^ Fourth, the NVM’s advantage against standard algorithms was observed across different manufacturers and field strengths, respectively, indicating that its specific approach accounts rather more for the observed improvement than scanner hardware. Finally, because NVM is a post-processing pipeline, it allows incorporation into emerging multi-parametric frameworks such as MRI-based RANO 2.0 and PET-RANO 1.0 criteria to provide complementary vascular information alongside various imaging modalities capturing both perfusion and metabolism features of malignant gliomas.^32–34^ Preliminary studies combining sequential GRE-DSC perfusion MRI with FET-PET have already demonstrated superior discrimination of true tumor progression from treatment-related changes.^35^ However, to date, harmonization of MRI- and PET-based data remains a critical issue in neuro-oncology.^36^

Addressing the NVM’s evidence gap, coordinated multi-center trials are required that validate its output against independent biological standards, ideally by co-registering voxel-wise perfusion maps with stereotactic biopsies or vessel-size imaging, to determine whether the observed numerical gains truly reflect microvascular biology.^13^ Harmonized GRE-DSC protocols across 1.5 T and 3.0 T platforms, augmented by optional dual-echo acquisitions, would clarify how far the model can narrow the residual vessel-size bias noted in consensus guidelines.^26^ A further priority is to test the clinical utility of the NVM-specific oxygenation metrics (CTH, OEF, CMRO_2_) in longitudinal glioma cohorts that include post-therapy scans and *IDH*-mutant tumors. Finally, algorithmic refinements, such as incorporating bidirectional leakage models or adaptive AIF selection, may further reduce residual bias in perfusion estimates and facilitate fully automated, vendor-neutral deployment in routine neuro-oncology practice.

Several methodological constraints limit the interpretation of our findings. First, this was a single-center, retrospective study exclusively applying GRE-DSC perfusion MRI. The known vessel-size bias of GRE-DSC and its greater susceptibility to macroscopic artefacts at 3.0 Tesla remain only partially mitigated by the NVM and cannot be eliminated without spin-echo or dual-echo acquisitions.^22^^,^^23^^,^^25^^,^^37^ Second, although NVM’s Bayesian fit probably elevates CBF, CBV and MTT, no external gold standard (ie vessel-size imaging or quantitative histology) was available to confirm that these calculated numerical gains are indeed biologically more accurate.^11^^,^^12^ Third, leakage-correction may inflate NVM values, although its impact is likely modest given the preload dose, contralateral normalization, and the finding that the largest gains occurred in CBF and MTT, indices largely unaffected by leakage after preload.^27^ In this context, by design, we did neither implement research grade leakage corrected SVD comparators nor automatic volumetric tumor segmentation. Both would redefine the estimand and endpoints from hotspot conspicuity to volume wide descriptors. Fourth, the broader dynamic range of NVM color-coded maps, combined with each pipeline’s distinctive texture and fixed window/level preset, introduces a risk of classification bias unrelated to physiology. Although inter-reader agreement for manual ROI placement was excellent, the use of circular ROIs still samples only a small portion of these highly heterogeneous tumors. Finally, the study was limited to pre-treatment *IDH*-wild-type glioblastomas; applicability to post-therapy assessment or other molecular subtypes and/or *IDH*-mutant gliomas remains to be verified in prospective cohorts.

In conclusion, in this GRE-DSC MRI perfusion study of patients with untreated *IDH*-wild-type glioblastoma NVM post-processing yielded consistently higher normalized CBF, CBV and MTT values than two widely used vendor pipelines, particularly when applying 3.0 Tesla. We emphasize that our claims are confined to relative improvements over conventional vendor processing within the stated design and do not imply biological superiority of any map. But these findings underscore the promise of model-based vascular analysis for routine perfusion MRI; however, the approach inherits the echo-regime bias of GRE-DSC data and remains partly susceptible to leakage effects and AIF selection. Prospective, multi-center studies with histopathologic correlation are therefore required before NVM metrics may qualify as quantitative markers.

## Data Availability

Data reported in this article can be shared in compliance with current data protection regulations by the European Union and approval from the relevant regulatory authorities. All proposals should be directed to the corresponding author.

## References

[1] Shiroishi MS , Castellazzi G, Boxerman JL, et al Principles of T2 *-weighted dynamic susceptibility contrast MRI technique in brain tumor imaging. J Magn Reson Imaging. 2015;41:296-313. 24817252 10.1002/jmri.24648

[2] Meier P , Zierler KL. On the theory of the indicator-dilution method for measurement of blood flow and volume. J Appl Physiol. 1954;6:731-744. 13174454 10.1152/jappl.1954.6.12.731

[3] Jespersen SN , Østergaard L. The roles of cerebral blood flow, capillary transit time heterogeneity, and oxygen tension in brain oxygenation and metabolism. J Cereb Blood Flow Metab. 2012;32:264-277. 22044867 10.1038/jcbfm.2011.153 PMC3272609

[4] Law M , Yang S, Babb JS, et al Comparison of cerebral blood volume and vascular permeability from dynamic susceptibility contrast-enhanced perfusion MR imaging with glioma grade. AJNR Am J Neuroradiol. 2004;25:746-755. 15140713 PMC7974484

[5] Hu LS , Baxter LC, Smith KA, et al Relative cerebral blood volume values to differentiate high-grade glioma recurrence from posttreatment radiation effect: direct correlation between image-guided tissue histopathology and localized dynamic susceptibility-weighted contrast-enhanced perfusion MR imaging measurements. AJNR Am J Neuroradiol. 2009;30:552-558. 19056837 10.3174/ajnr.A1377 PMC7051449

[6] Emblem KE , Mouridsen K, Bjornerud A, et al Vessel architectural imaging identifies cancer patient responders to anti-angiogenic therapy. Nat Med. 2013;19:1178-1183. 23955713 10.1038/nm.3289 PMC3769525

[7] Chida D , Okita Y, Utsugi R, et al Dynamic susceptibility contrast‑enhanced perfusion magnetic resonance imaging parameters for predicting MGMT promoter methylation and prognostic value in newly diagnosed patients with glioblastoma. Oncol Lett. 2024;28:610. 39493435 10.3892/ol.2024.14741 PMC11528182

[8] Law M , Young RJ, Babb JS, et al Gliomas: predicting time to progression or survival with cerebral blood volume measurements at dynamic susceptibility-weighted contrast-enhanced perfusion MR imaging. Radiology. 2008;247:490-498. 18349315 10.1148/radiol.2472070898 PMC3774106

[9] Patel P , Baradaran H, Delgado D, et al MR perfusion-weighted imaging in the evaluation of high-grade gliomas after treatment: a systematic review and meta-analysis. Neuro Oncol. 2017;19:118-127. 27502247 10.1093/neuonc/now148 PMC5193025

[10] Wu O , Østergaard L, Weisskoff RM, Benner T, Rosen BR, Sorensen AG. Tracer arrival timing-insensitive technique for estimating flow in MR perfusion-weighted imaging using singular value decomposition with a block-circulant deconvolution matrix. Magn Reson Med. 2003;50:164-174. 12815691 10.1002/mrm.10522

[11] Mouridsen K , Friston K, Hjort N, Gyldensted L, Østergaard L, Kiebel S. Bayesian estimation of cerebral perfusion using a physiological model of microvasculature. Neuroimage. 2006;33:570-579. 16971140 10.1016/j.neuroimage.2006.06.015

[12] Mouridsen K , Hansen MB, Østergaard L, Jespersen SN. Reliable estimation of capillary transit time distributions using DSC-MRI. J Cereb Blood Flow Metab. 2014;34:1511-1521. 24938401 10.1038/jcbfm.2014.111 PMC4158667

[13] Østergaard L , Jespersen SN, Mouridsen K, et al The role of the cerebral capillaries in acute ischemic stroke: the extended penumbra model. J Cereb Blood Flow Metab. 2013;33:635-648. 23443173 10.1038/jcbfm.2013.18 PMC3652700

[14] Yang X , Hu C, Xing Z, et al Prediction of ki-67 labeling index, ATRX mutation, and MGMT promoter methylation status in IDH-mutant astrocytoma by morphological MRI, SWI, DWI, and DSC-PWI. Eur Radiol. 2023;33:7003-7014. 37133522 10.1007/s00330-023-09695-w

[15] Park JE , Kim HS, Kim N, et al Prediction of pseudoprogression in post-treatment glioblastoma using dynamic susceptibility contrast-derived oxygenation and microvascular transit time heterogeneity measures. Eur Radiol. 2024;34:3061-3073. 37848773 10.1007/s00330-023-10324-9

[16] Louis DN , Perry A, Wesseling P, et al The 2021 WHO classification of tumors of the Central nervous system: a summary. Neuro Oncol. 2021;23:1231-1251. 34185076 10.1093/neuonc/noab106 PMC8328013

[17] Orsingher L , Piccinini S, Crisi G. Differences in dynamic susceptibility contrast MR perfusion maps generated by different methods implemented in commercial software. J Comput Assist Tomogr. 2014;38:647-654. 24879459 10.1097/RCT.0000000000000115

[18] Hansen MB , Tietze A, Kalpathy-Cramer J, et al Reliable estimation of microvascular flow patterns in patients with disrupted blood-brain barrier using dynamic susceptibility contrast MRI. J Magn Reson Imaging. 2017;46:537-549. 27902858 10.1002/jmri.25549

[19] Hales PW , d'Arco F, Cooper J, et al Arterial spin labelling and diffusion-weighted imaging in paediatric brain tumours. Neuroimage Clin. 2019;22:101696. 30735859 10.1016/j.nicl.2019.101696 PMC6365981

[20] Calamante F , Yim PJ, Cebral JR. Estimation of bolus dispersion effects in perfusion MRI using image-based computational fluid dynamics. Neuroimage. 2003;19:341-353. 12814584 10.1016/s1053-8119(03)00090-9

[21] Jackson A , Kassner A, Annesley-Williams D, Reid H, Zhu XP, Li KL. Abnormalities in the recirculation phase of contrast agent bolus passage in cerebral gliomas: comparison with relative blood volume and tumor grade. AJNR Am J Neuroradiol. 2002;23:7-14. 11827869 PMC7975509

[22] Kiselev VG , Novikov DS. Transverse NMR relaxation as a probe of mesoscopic structure. Phys Rev Lett. 2002;89:278101. 12513247 10.1103/PhysRevLett.89.278101

[23] Boxerman JL , Hamberg LM, Rosen BR, Weisskoff RM. MR contrast due to intravascular magnetic susceptibility perturbations. Magn Reson Med. 1995;34:555-566. 8524024 10.1002/mrm.1910340412

[24] Sugahara T , Korogi Y, Kochi M, Ushio Y, Takahashi M. Perfusion-sensitive MR imaging of gliomas: comparison between gradient-echo and spin-echo echo-planar imaging techniques. AJNR Am J Neuroradiol. 2001;22:1306-1315. 11498419 PMC7975222

[25] Troprès I , Grimault S, Vaeth A, et al Vessel size imaging. Magn Reson Med. 2001;45:397-408. 11241696 10.1002/1522-2594(200103)45:3<397::aid-mrm1052>3.0.co;2-3

[26] Boxerman JL , Quarles CC, Hu LS, et al; Jumpstarting Brain Tumor Drug Development Coalition Imaging Standardization Steering Committee Consensus recommendations for a dynamic susceptibility contrast MRI protocol for use in high-grade gliomas. Neuro Oncol. 2020;22:1262-1275. 32516388 10.1093/neuonc/noaa141 PMC7523451

[27] Boxerman JL , Schmainda KM, Weisskoff RM. Relative cerebral blood volume maps corrected for contrast agent extravasation significantly correlate with glioma tumor grade, whereas uncorrected maps do not. AJNR Am J Neuroradiol. 2006;27:859-867. 16611779 PMC8134002

[28] Pak E , Choi SH, Park C-K, et al Added value of contrast leakage information over the CBV value of DSC perfusion MRI to differentiate between pseudoprogression and true progression after concurrent chemoradiotherapy in glioblastoma patients. Investig Magn Reson Imaging. 2022;26:10-19.

[29] Tselikas L , Souillard-Scemama R, Naggara O, et al Imaging of gliomas at 1.5 and 3 tesla—A comparative study. Neuro Oncol. 2015;17:895-900. 25526734 10.1093/neuonc/nou332 PMC4483120

[30] Lu J , Li X, Li H. Perfusion parameters derived from MRI for preoperative prediction of IDH mutation and MGMT promoter methylation status in glioblastomas. Magn Reson Imaging. 2021;83:189-195. 34506909 10.1016/j.mri.2021.09.005

[31] Mannam SS , Nwagwu CD, Sumner C, Weinberg BD, Hoang KB. Perfusion-weighted imaging: the use of a novel perfusion scoring criteria to improve the assessment of brain tumor recurrence versus treatment effects. Tomography. 2023;9:1062-1070. 37368539 10.3390/tomography9030087 PMC10302394

[32] Wen PY , van den Bent M, Youssef G, et al RANO 2.0: update to the response assessment in neuro-oncology criteria for high- and low-grade gliomas in adults. J Clin Oncol. 2023;41:5187-5199. 37774317 10.1200/JCO.23.01059 PMC10860967

[33] Albert NL , Galldiks N, Ellingson BM, et al PET-based response assessment criteria for diffuse gliomas (PET RANO 1.0): a report of the RANO group. Lancet Oncol. 2024;25:e29-e41. 38181810 10.1016/S1470-2045(23)00525-9 PMC11787868

[34] Weller M , Le Rhun E, Van den Bent M, et al Diagnosis and management of complications from the treatment of primary central nervous system tumors in adults. Neuro Oncol. 2023;25:1200-1224. 36843451 10.1093/neuonc/noad038 PMC10326495

[35] Steidl E , Langen KJ, Hmeidan SA, et al Sequential implementation of DSC-MR perfusion and dynamic [(18)F]FET PET allows efficient differentiation of glioma progression from treatment-related changes. Eur J Nucl Med Mol Imaging. 2021;48:1956-1965. 33241456 10.1007/s00259-020-05114-0 PMC8113145

[36] Müller KJ , Forbrig R, Reis J, et al Measurable disease as baseline criterion for response assessment in glioblastoma: a comparison of PET -based (PET RANO 1.0) and MRI-based (RANO) assessments. Neuro Oncol. 2025;27:77-88. 39561103 10.1093/neuonc/noae208 PMC11726251

[37] Donahue KM , Krouwer HG, Rand SD, et al Utility of simultaneously acquired gradient-echo and spin-echo cerebral blood volume and morphology maps in brain tumor patients. Magn Reson Med. 2000;43:845-853. 10861879 10.1002/1522-2594(200006)43:6<845::aid-mrm10>3.0.co;2-j

